# Targeting the myeloid checkpoint receptor SIRPα potentiates innate and adaptive immune responses to promote anti-tumor activity

**DOI:** 10.1186/s13045-020-00989-w

**Published:** 2020-11-30

**Authors:** Tracy C. Kuo, Amy Chen, Ons Harrabi, Jonathan T. Sockolosky, Anli Zhang, Emma Sangalang, Laura V. Doyle, Steven E. Kauder, Danielle Fontaine, Sangeetha Bollini, Bora Han, Yang-Xin Fu, Janet Sim, Jaume Pons, Hong I. Wan

**Affiliations:** 1 ALX Oncology, Burlingame, CA USA; 2 Department of Pathology, University of Texas Southwestern Medical Center, Dallas, TX USA; 3 Present Address: Tallac Therapeutics, Burlingame, CA USA; 4 Present Address: Genentech, South San Francisco, CA USA; 5 Present Address: Coherus BioSciences, Redwood City, CA USA; 6 Present Address: ProLynx Inc., San Francisco, CA USA

**Keywords:** SIRPα, CD47, Macrophage, Phagocytosis, Immunotherapy, Innate immune checkpoint, Adaptive immunity

## Abstract

**Background:**

Signal regulatory protein α (SIRPα) is a myeloid-lineage inhibitory receptor that restricts innate immunity through engagement of its cell surface ligand CD47. Blockade of the CD47–SIRPα interaction synergizes with tumor-specific antibodies and T-cell checkpoint inhibitors by promoting myeloid-mediated antitumor functions leading to the induction of adaptive immunity. Inhibition of the CD47–SIRPα interaction has focused predominantly on targeting CD47, which is expressed ubiquitously and contributes to the accelerated blood clearance of anti-CD47 therapeutics. Targeting SIRPα, which is myeloid-restricted, may provide a differential pharmacokinetic, safety, and efficacy profile; however, SIRPα polymorphisms and lack of pan-allelic and species cross-reactive agents have limited the clinical translation of antibodies against SIRPα. Here, we report the development of humanized AB21 (hAB21), a pan-allelic anti-SIRPα antibody that binds human, cynomolgus monkey, and mouse SIRPα alleles with high affinity and blocks the interaction with CD47.

**Methods:**

Human macrophages derived from donors with various SIRPα v1 and v2 allelic status were used to assess the ability of hAB21 to enhance phagocytosis. HAB21_IgG subclasses were evaluated for targeted depletion of peripheral blood mononuclear cells, phagocytosis and in vivo efficacy in xenograft models. Combination therapy with anti-PD1/anti-PD-L1 in several syngeneic models was performed. Immunophenotyping of tissues from MC38 tumor-bearing mice treated with AB21 and anti-PD-1 was evaluated. PK, PD and tolerability of hAB21 were evaluated in cynomolgus monkeys.

**Results:**

SIRPα blockade with hAB21 promoted macrophage-mediated antibody-dependent phagocytosis of tumor cells in vitro and improved responses to rituximab in the Raji human tumor xenograft mouse model. Combined with PD-1/PD-L1 blockade, AB21 improved response rates by facilitating monocyte activation, dendritic cell activation, and T cell effector functions resulting in long term, durable antitumor immunity. In cynomolgus monkeys, hAB21 has a half-life of 5.3 days at 10 mg/kg and complete target occupancy with no hematological toxicity or adverse findings at doses up to 30 mg/kg.

**Conclusions:**

The in vitro and in vivo antitumor activity of hAB21 broadly recapitulates that of CD47 targeted therapies despite differences in ligand expression, binding partners, and function, validating the CD47–SIRPα axis as a fundamental myeloid checkpoint pathway and its blockade as promising therapeutic intervention for treatment of human malignancies.

## Background

The clinical development of immune checkpoint inhibitors (ICIs) has dramatically changed the landscape of cancer treatment [1–3]. ICIs targeting T-cell inhibitory receptors can induce complete and durable tumor immunity in patients with metastatic and treatment-refractory cancers. Despite the clinical promise of ICIs, only a fraction of patients within the ICI responsive cancer subtypes benefit from treatment with antibodies against PD-1, PD-L1, and CTLA4. Cancer is highly heterogeneous and complex and exploits a variety of mechanisms to evade immune surveillance beyond suppression of antitumor T cell responses [4]. Tumor-associated macrophages constitute a large fraction of the immune cell infiltrates within the tumor microenvironment of many human cancers [5]. Dendritic cells (DCs), although low in frequency within tumors, are potent and crucial mediators of antitumor immunity [6]. Given their prevalence and immunomodulatory activities, targeting regulators of macrophage and DC function is an attractive strategy to augment antitumor immunity and achieve additive or synergistic efficacy in combination with antitumor antibodies or ICIs.

Signal regulatory protein α (SIRPα) is an immunoinhibitory receptor expressed primarily by cells of the myeloid lineage including monocytes, macrophages, DCs, and neutrophils [7]. Upon interaction with its principal ligand, CD47, SIRPα transmits inhibitory signals that regulate DC homeostasis, self-recognition, and macrophage-mediated programmed cell removal [7, 8]. During malignant transformation, many human tumors exploit upregulation of CD47 thus activation of SIRPα signaling to avoid phagocytic clearance, resulting in the suppression of myeloid-mediated innate immunity and poor induction of antigen-specific immunity. SIRPα negatively regulates DC maturation, antigen presentation [9], and proinflammatory cytokine secretion [10]. In addition, it counteracts activating signals mediated by antibody engagement of Fcγ receptors (FcγR), which profoundly limits antibody-dependent cellular phagocytosis (ADCP) against tumors, restricts neutrophil transmigration [11], and maintains myeloid-derived suppressor cell (MDSC) functions [12]. Given the broad negative regulatory roles of SIRPα on innate immunity, a variety of CD47–SIRPα antagonists have been developed to promote the antitumor activity of phagocytes and myeloid cells. Blockade of the CD47–SIRPα interaction synergizes with both tumor-specific antibodies and ICIs by effectively reprogramming the myeloid compartment toward a proinflammatory phenotype improving tumor cell phagocytosis, antigen presentation, and T cell priming [9, 13]. A number of CD47 antagonists have entered the clinic with promising anticancer activity in both hematological and solid tumors [14–18].

Efforts to disrupt the CD47–SIRPα interaction have mainly focused on targeting CD47 due to its upregulation and ubiquitous expression on most human tumor types. However, CD47 is also broadly expressed on virtually all normal cells, including red blood cells and platelets, which creates a large antigen sink and CD47 blockers comprising an active Fc have shown dose-dependent cytopenia [19, 20]. We have previously shown that safety liabilities associated with CD47 blockers with active Fc domains can be overcome by eliminating Fc effector function [13, 16, 17, 21]. Targeting SIRPα is an orthogonal approach to inhibit the CD47–SIRPα pathway.

Herein, we explore pre-clinical pharmacology, pharmacokinetics, and exploratory safety associated with antibody-based blocking and antagonism of SIRPα. Utilizing an anti-SIRPα antibody we discovered in human antibody transgenic chickens [22], we demonstrate that pan-allelic anti-SIRPα antibody hAB21, which blocks the CD47–SIRPα interaction, broadly recapitulates the functional properties of CD47 antagonists by promoting the anticancer activity of both tumor-specific antibodies and ICIs in a macrophage and DC dependent manner. This leads to the induction of adaptive immunity, resulting in complete and durable antitumor responses in mice. HAB21 exhibits favorable safety and pharmacokinetic (PK) profiles in monkeys with no hematological or immune-related adverse events. Thus, targeting SIRPα with hAB21 is a promising therapeutic approach to treat human cancers by reprogramming innate immunity to sensitize cancers to immunotherapy.

## Methods

### Expression and purification

All antibodies were expressed in Expi293 cells (Invitrogen) using standard manufacturer’s protocol. Expression cultures were typically grown for 5 days at 37 °C in 8% CO_2_. Supernatants were harvested via centrifugation and sterile filtered. Proteins were affinity purified utilizing MabSelect Sure LX resin (GE Healthcare). For SPR screening, the IgV domains of human SIRPα v1 (NP_542970.1) and human SIRPα v2 (CAA71403.1) are expressed in Expi293 cells as described above as either a His-tagged or His-Avi-tagged fusions and purified using Ni-Sepharose 6 Fast Flow affinity purification and polished via gel filtration through a superdex hi-prep resin (GE Healthcare). The anti-SIRPα v1 specific antibody clone HEF-LB was generated as described in reference [23]. For crystallography, the IgV domain of SIRPα v1 and Fab fragments was generated as described [14].

### Humanization of antibodies

Parental clones of hAB21 (AB21 and AB25) were isolated from SynVH, a transgenic chicken, and have fully human heavy chains but chicken light chains [14]. In order to humanize the chicken-derived light chains, chicken hypervariable region (HVRs) of AB25 were grafted onto human lambda light chain IGLV1, IGLV2 and IGLV3 frameworks, and chicken HVRs of hAB21 were only grafted onto human lambda light chain IGLV1 framework [24]. Combination of antibodies was generated from 4 humanized light chains with two heavy chains (derived from AB21 and AB25 respectively) in Expi293, purified and tested for SIRP binding. The top humanized antibody, with favorable expression and binding properties, was designated hAB21 and selected for further testing.

### Determination of ***K***_D_

The binding affinities of AB21 and hAB21 to human SIPRα v1 and v2 were determined using direct immobilization of the antibodies (via GLC chip). All antibodies and proteins were used at their nominal concentrations determined by A280 absorbance and molar extinction coefficient. Analytes (human SIRPα v1 and v2) were injected in a “one-shot” kinetic mode and flowed over AB21 and hAB21 immobilized (~ 1000 RUs) on GLC chips using ProteOn Amine Coupling Kit. For the immobilization step, GLC chip was activated with EDAC/Sulpho-NHS 1:1 (Biorad) diluted 1/100 for 300 s at 25 μL/min. AB21 and hAB21 were diluted to 80 nM concentration in 10 mM sodium acetate buffer pH 4.5 and immobilized to the chip at 30 μL/min for 50 s. Chip was inactivated with ethanolamine for 300 s at 25 μL/min. The analytes (human SIRPα v1 and v2 protein) were injected in a “one-shot” kinetic mode at nominal concentrations of 10, 3.3, 1.1, 0.37, 0.12 and 0 nM. Association times were monitored for 90 s at 100 μL/min, and dissociation times were monitored for 1200 s. The surfaces were regenerated with a 2:1 v/v blend of Pierce IgG elution buffer/4 M NaCl. Biosensor data were double-referenced by subtracting the interspot data (containing no immobilized protein) from the reaction spot data (immobilized protein), and then subtracting the response of a buffer “blank” analyte injection from that of an analyte injection. Double-referenced data were fit globally to a simple Langmuir model and the *K*_D_ value was calculated from the ratio of the apparent kinetic rate constants (*K*_D_ = *k*_d_/*k*_a_).

### Crystallization of anti-SIRPα Fab: SIRPα complexes

A pure sample of anti-SIRPα Fab-SIRPα V1 complex at a concentration of 11.3 mg/mL in a buffer of 10 mM Tris pH 7.4, 50 mM NaCl was set for sitting drop vapor diffusion with sparse matrix crystallization screen kits available Qiagen. The condition and crystal form that gave quality diffraction leading to a complete dataset was 1. 0.1 M Sodium Acetate pH 4.0, 0.2 M Ammonium Sulfate, 18% (w/v) PEG 4000 (Cryo-protectant: 5% v/v Ethylene Glycol). Even though the crystallization condition contained a high percentage of PEG 4000, ethylene glycol was added as cryo-protectant to protect the crystal form from deteriorating and/or forming ice during freezing. These samples were screened for protein X-ray diffraction at the NSLS-II 17-ID AMX beamline; yielding a dataset with diffraction of 2.27 Angstroms resolution. Further crystallography data collected are detailed in Additional file 1: Table S1. Structure PDB ID: 7KPG.

### Cell binding and blockade of CD47 binding to SIRPα cells

For detection of cell binding, AB21 was fluorescently labeled with the Alexa Fluor 647 Protein Labeling Kit (Thermo Fisher Scientific) according to the manufacturer’s instructions. 250,000 cells per well in staining buffer (PBS, 0.5% BSA or 2% FBS) were plated in 96-well plates (Falcon). Cells were first stained with fixable Live/Dead Stain (Invitrogen) and washed once in staining buffer prior to all binding assays.

To detect SIRPα binding to cells, 500 nM Alexa Fluor 647-labeled AB21 was titrated 1:4 for seven dilutions and added to cells in 100 μL volume of FACS buffer (PBS + 0.5% BSA) supplemented with a cocktail of human Fc block (Miltenyi Biotec) or mouse Fc block (Biolegend), anti-CD14 (Biolegend) for human and cynomolgus PBMCs or anti-CD11b (Biolegend) for mouse splenocytes. After a 60-min incubation on ice, cells were washed twice in staining buffer and fixed in 0.5% paraformaldehyde.

To block CD47 binding to SIRPα on PBMCs, Alexa Fluor 647 labeled CD47Fc at a concentration of 500 nM and 1:4 titration starting at 1 µM of AB21 were added to cells. After a 60-min incubation on ice, cells were washed twice in staining buffer and fixed in 0.5% formaldehyde.

Cells were analyzed on a FACS Canto II (BD Biosciences), with subsequent data analysis using Flowjo 10.7.

### Cell lines

4T1, CT26, Raji, MDA-MB-231 and DLD-1 cells were obtained from the American Type Culture Collection (ATCC). MC38 cells were obtained from MuriGenics. All cell lines were cultured according to standard protocols.

### Animals

All mouse experiments except for MDA-MB-231 and batf3 KO mice were conducted according to protocols approved by Institutional Animal Care and Use Committee (IACUC) of ALX Oncology. Tumor model with MDA-MB-231 and batf3 KO mice were conducted in compliance with UTSW Human investigation and UTSW Institutional Animal Care and Use Committee protocols. Female *Batf3*^−/−^ mice in the C57BL6/J background and NSG-SMG3 mice were purchased from The Jackson Laboratory. Female BALB/c, C57BL/6 and NOD-SCID animals, age 6–8 weeks old, were purchased from Charles River Laboratories International (Hollister, CA). All animals were housed according to institutional IACUC guidelines.

Female naïve cynomolgus monkeys (*Macaca fascicularis*) were provided by Charles River Laboratories. The in-life portion of the study was conducted by CRL’s testing facility (Reno NV), and the animals were released to the CRL testing facility’s colony at the end of the study.

### Generation of monocyte-derived macrophages and phagocytosis assay

Human CD14^+^ cells were purified from Trima residuals (Vitalant) with Ficoll-Paque Plus and negative selection (Monocyte Isolation Kit II, Miltenyi Biotec) according to the manufacturers’ protocols. Monocyte-derived macrophages (MDM) were made by seeding 10 million CD14^+^ cells into 150 mm tissue culture dishes (Corning) in growth medium supplemented with 10% human AB serum (Corning) or 10% FBS and 50 ng/mL MCSF. Cells were cultured for 7–11 days. Adherent cells were detached from culture plates with TrypLE Select (Thermo Fisher Scientific). Target cells (DLD-1) were labeled with the Celltrace CFSE Cell Proliferation kit (Thermo Fisher Scientific) according to the manufacturer’s instructions. 100,000 target cells and 50,000 MDMs were incubated in ultra-low attachment U-bottom 96-well plates (Corning) with anti-SIRPα antibodies and the corresponding tumor-specific antibody for 2 h at 37 °C. Cetuximab was added at a concentration of 0.01–0.1 µg/mL.

For flow cytometry, cells were incubated in human FcR blocking reagent (Miltenyi Biotec) and stained with fluorochrome-labeled antibodies against CD33 (clone WM53, Biolegend) and CD206 (clone 15–2, Biolegend). To eliminate macrophage/target cell adhesion from analyses, antibody against CD326 (clone 9C4, Biolegend) was included. Furthermore, a pulse geometry gate of forward scatter signal area vs height was used to select for single cells. Fixable viability dye (Thermo Fisher Scientific) was used to identify live cells. Cells were acquired on a FACS Canto II flow cytometer (BD Biosciences) with subsequent analysis using FlowJo software. Percent phagocytosis indicates the percentage of viable CD33+ CD206+ macrophages that stain negative for CD326 and positive for CFSE. Where applicable, 4 parameter fit curves were generated with Prism 7 software (GraphPad).

### In vitro PBMC culture

Peripheral blood mononuclear cells (PBMC) were isolated from Trima residuals of healthy individuals with Ficoll-Paque Plus. 500,000 PBMCs were incubated in U-bottom 96-well plates (Falcon) with anti-SIRP at a concentration of 10 ug/mL for 48 h at 37C.

For quantification of PBMC subsets by flow cytometry, cells were incubated in human FcR blocking reagent and stained with a cocktail of fluorochrome-labeled antibodies against lin—(CD3, CD14, CD16, CD19, CD56) and HLADR. Fixable viability dye was used to identify live cells. After staining, cells were washed and fixed with 0.5% paraformaldehyde in PBS. Prior to acquisition, absolute counting beads (Thermo Fisher) were added and samples were acquired with Canto II flow cytometer and analyzed using FlowJo software.

### Tumor studies

Isoflurane anesthesia was used on mice to eliminate or minimize pain and distress during tumor implantation. 2 × 10^6^ MC38, 2 × 10^6^ CT26, 5 × 10^5^ B16F10 and 5 × 10^5^ 4T1 cells were resuspended in 100ul of PBS or RPMI and implanted subcutaneously into the flank of female C57BL/6 mice for MC38 and B16F10 and BALB/c for CT26 and 4T1. 5 × 10^6^ Raji cells were resuspended in 100 ul 1:1 PBS:Matrigel (Corning) and implanted subcutaneously in the flank of NOD-SCID mice.

When tumors reached an average of 50-180mm^3^, as calculated with the formula volume = (width^2^ × length)/2, mice were randomized into treatment groups. All treatments were dosed intraperitoneally (i.p.) or intratumorally. For xenograft models in NOD-SCID mice, rituximab was dosed at 3 mg/kg and AB21 at 10 mg/kg, five times every 3 days. In syngeneic models, mice were treated with 10 mg/kg for CT26, MC38, B16F10 or 30 mg/kg for 4T1 of clone AB21, 10 mg/kg anti-PD-1 (BioXcell, clone RMP1-14) and 2 mg/kg (MC38 models) anti-PD-L1 (murine IgG1, ALX Oncology). For intratumoral injections, 50 µg of AB21 was given four times every 3 days.

For re-challenge experiments, treated mice with complete tumor eradications were re-challenged with either 2 × 10^6^ MC38 cells on one flank or 0.5 × 10^6^ B16F10 tumor cells on the opposite flank. Age-matched naïve mice were used as control.

For cellular depletion experiments, mice were dosed intraperitoneally with 250 ug of either anti-CSF1R (BioXcell, clone AFS98), anti-CD8*b* (Bioxcell, clone 53-5.8) or anti-GR1 (BioXcell, clone RB6-8C5) on days 2, 5, 10 and 15 post-tumor implantation. Confirmation of cellular depletion was performed on tumor and spleen of spare cellular depleted mice on days 1, 3, 4 and 8 post-injection by flow cytometry using anti-CD45 (Biolegend, clone 30-F11), anti-CD3 (Biolegend, clone 145-2C11), anti-CD8 (Biolegend, clone 53.6.7), anti-CD11b (ebioscience, clone M1/70), anti-Ly6C (Biolegend, clone 145-2C11), MHCII (ebioscience, clone M5/114.15.2) and F4/80 (Biolegend, clone BM8).

Humanized mice were generated with 4-week NSG-SGM3 female mice and irradiated with 100 cGy (X-ray irradiation with X-RAD 320 irradiator) 1 day prior to CD34+ cells transfer. Irradiated mice were treated with Bactrim (Aurora Pharmaceutical LLC) water for 2 weeks. Cord blood was obtained from UT Southwestern (UTSW) Parkland Hospital. Human CD34+ cells were purified from cord blood by density gradient centrifugation (Ficoll® Paque Plus, GE healthcare) followed by positive immunomagnetic selection with anti-human CD34 microbeads (Stem Cell). 10^5^ CD34+ cells were intravenously injected into recipient mice.

12 weeks after engraftment, humanized mice with over 40% human CD45^+^ cells reconstitution and age and sex matched non-humanized mice were inoculated with 2 × 10^6^ MDA-MB-231 tumor cells subcutaneously on the right flank. Tumor volumes were measured by length (*a*), width (*b*) and height (*c*) and calculated as tumor volume = *abc*/2. 8 days later when tumors were around 50 mm^3^, mice were intratumorally treated with 50 µg AB21, HEF-LB or PBS, four time every 3 days.

### 4T1 Lung metastasis quantification

BALB/c mice were implanted with 4T1 cells and lungs were harvested 8–9 days post-last injection for metastatic nodule quantification. In brief, lungs were harvested in ice-cold 1xPBS, minced into small pieces then transferred into digestion solution consisting of 2 mg/mL collagenase type V (Worthington) supplemented with 50 ug/mL DNAse (Sigma) and incubated for 2 h in a 37C incubator with end-over-end rotation. Suspension was transferred into 70-um strainer, washed once in 1 × PBS then transferred into 10 mL selection media consisting of RMPI 1640 supplemented with 10% FBS, penicillin–streptomycin and 10 ug/mL 6-thioguanine (Sigma). Three to four 1:10 serial dilutions were plated either in 6-well plates or 10-cm dishes and cultured for 10–14 days at 37C, 5% CO_2_. Metastatic plaques were then fixed in methanol for 5 min at room temperature, re-hydrated in distilled water then stained with 0.03% methylene blue (Sigma) for 5 min at room temperature. Dye was then discarded, plate was rinsed gently with distilled water and allowed to air-dry prior to counting plaques.

### Immunophenotyping

For immune response monitoring in tumor-bearing mice, anti-SIRPα was dosed either three times in combination with anti-PD-1, or twice with anti-SIRPα and once with anti-PD-1, all injections were administered i.p 3 days apart at 10 mg/kg.

Spleens and tumors were harvested either 2 or 3 days post-last injection for immunophenotyping. Spleens were processed into single-cell suspension in ice-cold PBS, lysed with ACK lysis buffer (Gibco), washed twice and re-suspended in PBS supplemented with 2% FBS. Tumor-derived single-cell suspensions were prepared using a cocktail of Collagenase I (Worthington), Collagenase IV (Sigma) and DNAse (Sigma) for 45 min at 37° C. Cell counts were performed using ViCell counter (Beckman Coulter) for spleen and lymph node and trypan blue exclusion with hemocytometer for tumor. Aliquots of 1–2 10^6^ cells were either used for cell-surface antigen staining or stimulation for cytokine assessment. For surface staining, cells were stained with LIVE/DEAD fixable dye (Thermo Fisher), followed by mouse Fc-block (Biolegend) and subsequently stained with antibodies according to cell-type specific antibody panels for at least 30 min at 4 °C. CD4 clone GK1.5, CD8 clone 53-6.7, CD25 clone PC61, CD3 clone 145-2C11, CD45 clone 30-F11, CD47 clone MIAP301, NKp46 clone 29A1.4, PD1 clone J43, FoxP3 clone FJK-16s, Ki67 clone SolA15, Granzyme B clone QA16A02, CD44 clone IM7, CD62L clone MEL-14, TNFa clone MP6-XT22, IFNg clone XMG1.2, SIGLEC H clone 551, CCR7 clone 4B12, CD172 clone P84, CD86 clone GL-1, MHCII clone M5/114.15.2, GR-1 clone RB6-8C5, 33D1 clone 33D1, CD11b clone M1/70.15, CD11c clone N418, CD103 clone 2E7, Ly6C clone NH1.4, F4/80 clone BM8, CD24 clone M1/69. All flow antibodies were purchased from either Biolegend or Thermo Fisher.

For PMA/ionomycin ex-vivo stimulation to quantitate IFNγ^+^ cells, total splenic and tumor cells were plated at 1 × 10^6^ cells/well in complete RPMI 1640 comprised of 10% heat-inactivated FBS, 2% Pen/Strep, 1% Glutamax, 1% MEAA, 1% sodium pyruvate, 25 mM HEPES and 5 μM ß-mercaptoethanol supplemented with 50 ng/ mL PMA (Fisher Scientific) and 1 μM ionomycin (Sigma) in the presence of Golgi-Stop for at least 4 h at 37 °C, 5% CO_2_, and subsequently stained with antibodies to surface and intracellular markers. Samples were then acquired using Attune NxT (Thermo Fisher) or Canto II (BD). Analysis was performed using FlowJo 10.0 (BD) and tabulated using GraphPad Prism 7.3.

### HAB21 and soluble SIRPα serum ELISA

Immulon 96-well ELISA plates (Thermo Fisher Scientific, 3855) were coated overnight with human wild type SIRPα, variant 1 (ALX Oncology) in PBS. Plates were washed with Tris-Buffered Saline Tween-20 (TBST, 25 mM Tris, 0.15 M NaCl, 0.05% Tween-20, pH 7.5) and blocked for 1 h with assay buffer (PBS, 1% BSA, 0.05% Tween-20, 0.25% CHAPS, 5 mM EDTA, 0.35 M NaCl). Serum samples diluted a minimum of 1:50 in assay buffer or hAB21 standard curve protein (two-fold serial dilutions from 160 to 1.25 ng/mL, in 1:50 normal cyno serum diluted in assay buffer) were added to blocked plates for 1 h. Plates were washed with TBST. Standard curves and samples were incubated for 1 h with biotinylated goat anti-human IgG (H + L) antibody (Bethyl, A80-319B), washed with TBST, incubated for 30 min with HRP-conjugated Avidin D (Vector, A2004), and washed with TBST. All plates were incubated with 1-Step Ultra TMB ELISA solution (Thermo Fisher Scientific, 34028) and the reaction was stopped with 0.16 M sulfuric acid solution (Thermo Fisher Scientific, N600). Plates were read at an O.D. of 450 nm with a background reference reading at 570 nm on a SpectraMax i3 plate reader (Molecular Devices). Protein concentrations of serum samples were interpolated from the hAB21 standard curve with a 4-parameter fit curve using Prism software (GraphPad).

For the detection of soluble SIRPα, similar assay to hAB21 serum ELISA was performed except for the following. 2 ug/mL anti-SIRPα mouse IgG1 antibody (AB136b, ALX Oncology, a non-CD47 blocking antibody that binds both cyno and human SIRPα with high affinity) in PBS was used to coat the plates. Cyno PK serum samples diluted 1:25 in assay buffer or SIRPα standard curve protein (ALX135, ALX Oncology, 25 to 0.024 ng/mL, diluted in assay buffer) were added to blocked plates for 1 h. Pre-dose cynomolgus serum samples spiked with or without 200 ug/mL of anti-SIRPα human IgG1 antibody were run as controls since most of the PK samples contained at least 200 ug/mL anti-SIRPα human IgG1 antibody. After incubating samples for 1 h in the ELISA, plates were washed with TBST. 5 ug/mL anti-SIRPα human IgG1 kick-off Fab + 6 × His tag (AB115f, ALX Oncology, binds both cynomolgus and human SIRPα with high affinity) was added to samples for 1 h. Plates were washed with TBST. Samples were incubated for 1 h with 0.15 ug/mL rabbit anti-6 × His Tag HRP conjugated antibody (abcam cat. # ab1187).

### HAB21 receptor occupancy

For the analysis of receptor occupancy, 75 μL aliquots of whole blood sample were washed in 1.5 mL FACS buffer (PBS, Thermo Fisher Scientific + 0.5% bovine serum albumin, Sigma) and stained with fluorochrome-conjugated antibodies against CD14 (M5E2, Biolegend) and HLA-DR (L243, Biolegend), fixable viability dye eFluor 506 (Thermo Fisher Scientific), and FcR blocking reagent, human (Miltenyi Biotec). SIRPα occupancy was detected using labeled SIRPα antibodies which would compete with test article for binding to SIRPα. For detection of SIRPα occupancy, Alexa-Fluor 647 conjugated (Thermo Fisher Scientific) hAB21 was added to the lineage stains and cells were incubated at 4 °C for 1 h. All stains were then washed in 1.5 mL FACS buffer and erythrocytes were lysed in 1.5 mL FACS lysing solution (BD Biosciences) for 10 min at room temperature. Cells were washed in 1.5 mL FACS buffer and resuspended in FACS buffer. Cells were analyzed by flow cytometry on a FACS Canto II (BD Biosciences).

Data were analyzed with FlowJo 10.4 software (Becton Dickinson). To measure SIRPα occupancy on monocytes, geometric mean fluorescence intensity (MFI) in the Alexa-Fluor 647 channel was determined for CD14^+^HLADR^+^ cells. To calculate percent SIRPα occupancy, this value was normalized to the MFI for the predose timepoint as follows: MFI was calculated as a percentage of MFI at the predose timepoint. This number was subtracted from 100%, with the result being the percent occupancy. Thus, MFI greater than or equivalent to the predose level is reported as zero percent occupancy and MFI equivalent to background is reported as 100% occupancy. All samples for which SIRPα staining was equivalent to or greater than staining of predose samples were considered to have no occupancy.

### Cynomolgus monkey study

The in-life of monkey study was conducted by Charles River Laboratories (Reno, NV). The study was performed in accordance with the standard operating procedures and Good laboratory Practices (GLP). Male cynomolgus monkeys (*n* = 2/group) of Chinese origin were administered hAB21 on Days 1 and 8 for a total of 2 intravenous doses at doses 10 and 30 mg/kg by slow bolus IV injection. Hematology was assessed by Charles River Laboratories and were collected from all animals on Days—4, 4, 8, 15, and 22. For PK bioanalysis, serum was collected from animals at the following time points: 0 (predose), 1, 3, 8, 24, 72, and 168 h postdose on Day 1. On Day 8, serum was collected from animals at the following time points: 1, 3, 8, 24, 72, 168 h, Day 18 and D22 postdose. For receptor occupancy assay, blood samples were drawn and collected for all animals at the following time points: predose (Day-4), 4, 24, and 72 h after the Day 1 dose; predose, 4, 24, 72, and 168 h after the Day 8 dose; Day 18 and Day 22.

## Results

**Fig. 1:**
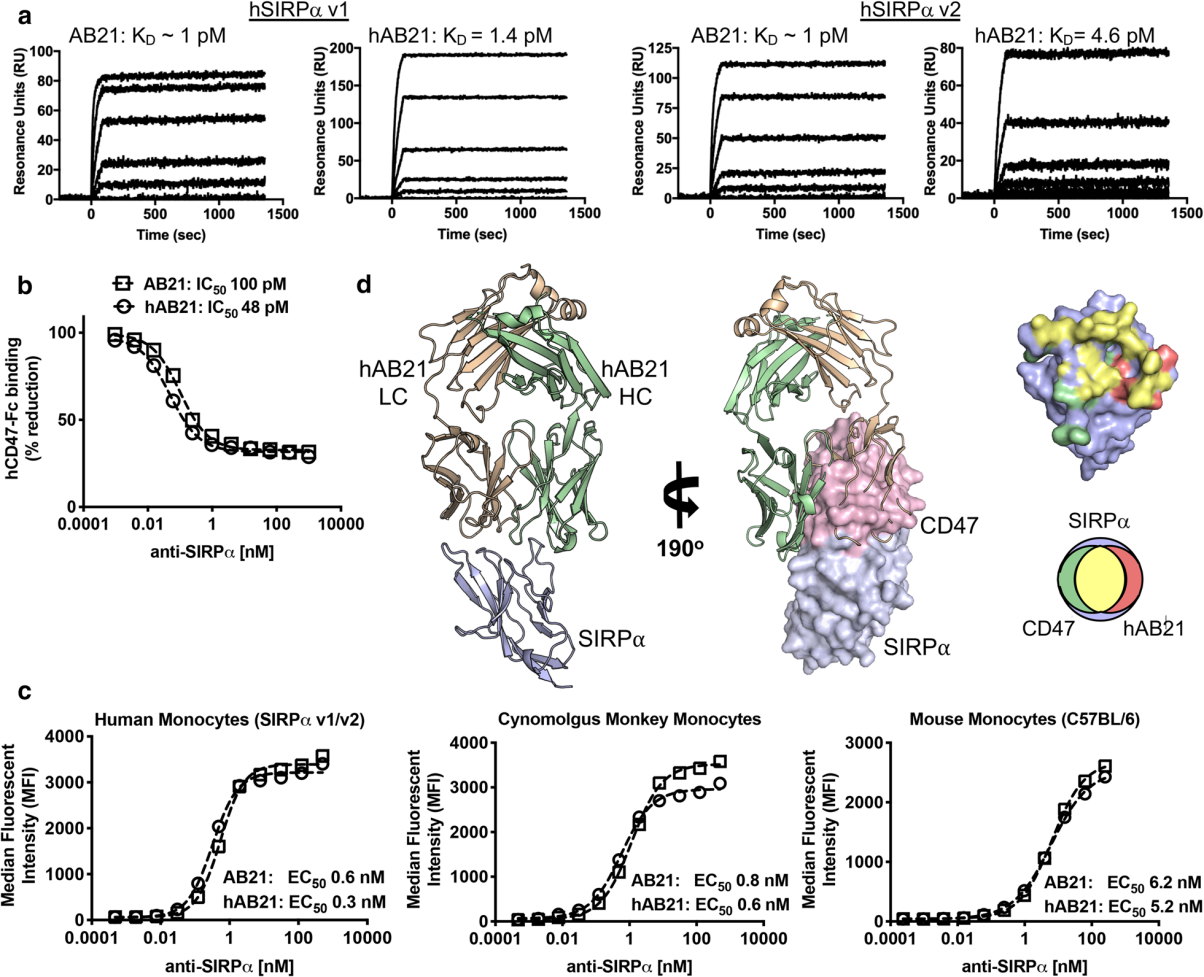
Pan-allelic and species cross-reactive antibody, hAB21, binds SIRPα with high affinity and blocks CD47–SIRPα interaction. **a** Binding affinities (pM) of AB21 and humanized hAB21 to human SIRPα v1 and v2 were determined by SPR. **b** Binding curves of hCD47 on CD14^+^ monocytes in the presence of titrated AB21 and hAB21. PBMCs were incubated with 500 nM fluorescent hCD47-Fc and increasing concentration of AB21 and hAB21 at 4 °C for 60 min, washed and evaluated by flow cytometry. Data are shown as ratio of CD14^+^ incubated with hCD47-Fc only. **c** Flow cytometry analysis of AB21 and hAB21 binding to human, cynomolgus and mouse CD14^+^ monocytes. Fluorescent-labeled AB21 and hAB21 were incubated in increasing concentration with cells at 4 °C for 60 min, washed and evaluated by flow cytometry. Mean fluorescent intensity is measured. **d** Crystal structure of hAB21 anti-SIRPα Fab bound to the Ig-V domain of human SIRPα V1 (purple). CD47 domain (pink) was superimposed onto the crystallized complex. The adjacent Venn diagram and surface map depict the epitopes bound by CD47 (green) and hAB21 (red); shared epitopes bound by both CD47 and hAB21 to SIRPα V1 are colored yellow accordingly

We previously described a family of anti-SIRPα antibodies that antagonize SIRPα through competition for the CD47-binding site located on the IgV domain of SIRPα [22]. The anti-SIRPα blocking antibody clone AB21 was discovered from immunized SynVH chickens, which contain human V_H_ immunoglobulin (Ig) repertoires paired with the natural chicken light chain repertoires [25]. The chimeric antibody AB21 binds to human SIRPα v1 and v2 alleles with picomolar affinity (Fig. 1a), potently blocks the interaction between CD47 and SIRPα (Fig. 1b), and cross-reacts with cynomolgus and murine SIRPα (Fig. 1c). Since clone AB21 contained the chicken light chain sequences, we carried out humanization as described in the Materials and Methods to derive the humanized version, named hAB21. Humanized AB21 (hAB21) retains the SIRPα binding and antagonistic properties of the parental AB21 (Fig. 1a–c). The structure of a Fab version of the antibody in complex with the IgV domain of human SIRPα v1 was solved by X-ray crystallography (Fig. 1d). The hAB21 Fab binds to SIRPα at an epitope highly overlapping with that of CD47, with the VL domain overlapping with the majority of the CD47 binding site while the VH domain CDR-H3 inserts into the central pocket of SIRPα that is crucial for CD47 binding, in agreement with the ability of hAB21 to completely block and antagonize the CD47–SIRPα interaction (Fig. 1d). AB21 and hAB21 were used interchangeably for subsequent experiments.

### Fc-domain IgG subclass influences the in vitro and in vivo activity of hAB21

Antibody IgG subclass dictates FcγR interactions that either promote (wild-type IgG1 and IgG4) or limit (IgG2Δa and IgG1^FcDead^) antibody effector functions [26–29]. Competition for FcγR binding between anti-SIRPα antibodies and anti-tumor antibodies may impact macrophage-mediated a ADCP. Therefore, we investigated the impact of IgG subclass on anti-SIRPα antibody-mediated enhancement of macrophage phagocytosis in vitro.

**Fig. 2:**
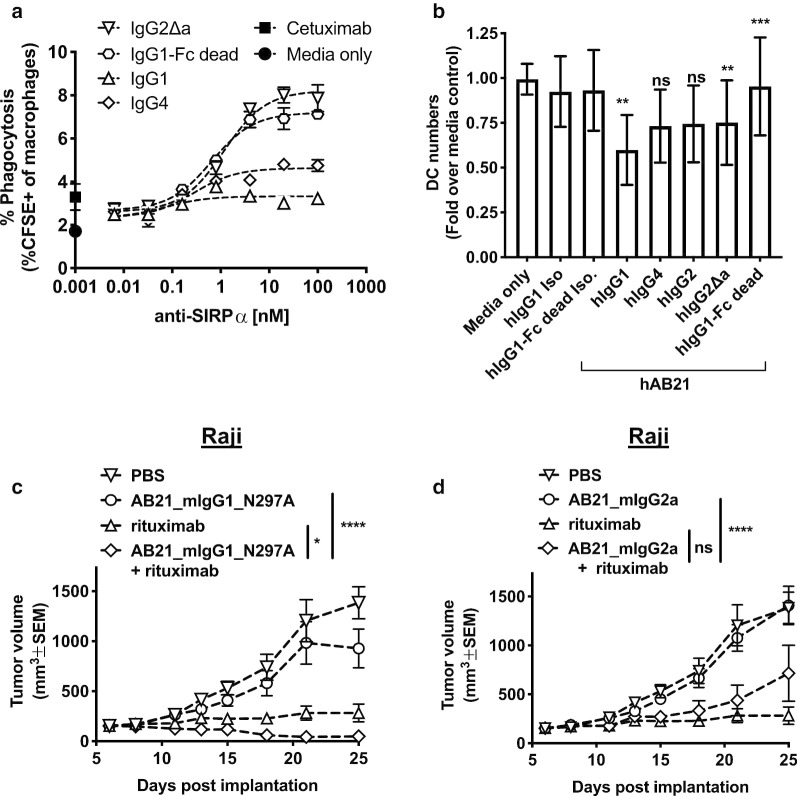
IgG subclass influences the efficacy of anti-SIRPα antagonist antibodies in vitro and in vivo*.* **a** In vitro phagocytosis experiment with human MDMs cultured in the presence of human AB serum and DLD-1 cells in the presence of cetuximab at a constant concentration and titrated anti-SIRPα antibodies with different IgG subclass. Percent of macrophages that engulfed tumor cells is indicated on the y-axis. On the y-axis, closed circle indicates background phagocytosis observed with media only and closed square indicates cells treated with cetuximab only. **b** Relative dendritic cell numbers in human PBMCs after 48-h incubation with hAB21-IgG variants. Each bar indicates relative DC number as compared to media only. Statistics were performed using One-Way ANOVA, Dunnett’s multiple comparisons. **c**, **d** Raji B-cell lymphoma cells were implanted subcutaneously on the right flanks of NOD-SCID mice. Mice with established tumors (average of 154 mm^3^) were randomized, *n* = 10/group, and treated intraperitoneally with vehicle, rituximab, AB21 mIgG1_N297A (inactive) (**c**), AB21 mIgG2a (active) (**d**), or AB21 inactive or active + rituximab. Mice were treated five doses every 3 days. Statistics were performed using Two-Way ANOVA, Tukey’s multiple comparisons test. *p < 0.05, **p < 0.01, ***p < 0.001 and ****p < 0.0001

HAB21 was expressed as either a human IgG1, IgG4, IgG2Δa, or IgG1^FcDead^ subclass and macrophage phagocytosis of DLD-1 tumor cells opsonized with the anti-EGFR antibody cetuximab alone or in combination with hAB21 was quantified by flow cytometry. Cetuximab alone promoted macrophage phagocytosis of DLD-1 tumors cells, and this was further enhanced in a dose-dependent manner by combination with hAB21, but only when the Fc-domain of hAB21 was engineered to minimize binding to FcγR (Fig. 2a). HAB21 with an inactive IgG1^FcDead^ or attenuated IgG2Δa Fc-domain enhanced cetuximab-mediated macrophage phagocytosis of DLD-1 tumor cells, whereas hAB21 with an active IgG1 or IgG4 Fc-domain demonstrated little to no impact on cetuximab-mediated phagocytosis (Fig. 2a). These results suggest that competition between hAB21 and cetuximab for macrophage FcγR limits the phagocytosis enhancing effects of anti-SIRPα antibodies or that hAB21 with an active IgG simultaneously co-engages SIRPα and FcγR on macrophages, resulting in a heterotrimeric interaction. This has been described as the “scorpion effect” [30] and this heterotrimer interaction would limit FcγR clustering by cetuximab, thereby decreasing pro-phagocytosis signals and reducing the enhancing effects of phagocytosis with hAB21 and cetuximab.

To further investigate the impact of IgG subclass on hAB21 activity, we cultured primary human peripheral blood mononuclear cells (PBMCs) with hAB21 containing different IgG subclasses for 48 h and quantified the numbers of T, B, monocytes, NK and DCs by flow cytometry. There was no significant change in the number of T, B, monocytes or NK cells (Additional file 1: Fig. S1A); however, PBMCs cultured with hAB21 containing an IgG1, IgG4, IgG2, or IgG2Δa Fc-domain had lower numbers of lineage negative (CD3^−^, CD19^−^, CD56^−^) HLA-DR^+^ DCs, whereas hAB21-IgG1^FcDead^ had limited impact on the numbers of respective DC lineage (Fig. 2b). Interestingly, although monocytes express higher levels of SIRPα than DCs (Additional file 1: Fig. S1B), we observed no changes in the number of monocytes present in PBMCs cultured with hAB21 irrespective of its IgG subclass.

Finally, we investigated the impact of Fc-domain IgG subclass on the in vivo antitumor activity of AB21 in NOD SCID mice bearing subcutaneous human CD20^+^ Raji tumor cells alone or in combination with rituximab. Treatment with rituximab alone substantially delayed tumor growth compared to the PBS control, whereas monotherapy with AB21 containing either an inactive (mouse IgG1 N297A) or active (mouse IgG2a) Fc-domain had minimal impact on tumor growth (Fig. 2c, d and Additional file 1: Fig. S2). The combination of AB21 containing an inactive Fc-domain with rituximab significantly improved anti-tumor activity compared to rituximab alone (Fig. 2c and Additional file 1: Fig. S2), consistent with the known impact of CD47–SIRPα antagonism on potentiating responses to antitumor antibody therapy [13, 31]. However, combination therapy using AB21 with an active Fc-domain decreased antitumor activity when compared to rituximab alone (Fig. 2d). Collectively, these data indicate that anti-SIRPα antibodies capable of triggering Fc effector functions limit the therapeutic activity both in vitro and in vivo through multiple mechanisms involving macrophage-mediated ADCP and DC depletion. We subsequently chose human IgG1^FcDead^ as the Fc-domain for hAB21 and mouse IgG1_N297A as the Fc-domain for AB21 for further experimentation.

### HAB21 potentiates macrophage phagocytosis irrespective of SIRPα genotype

**Fig. 3:**
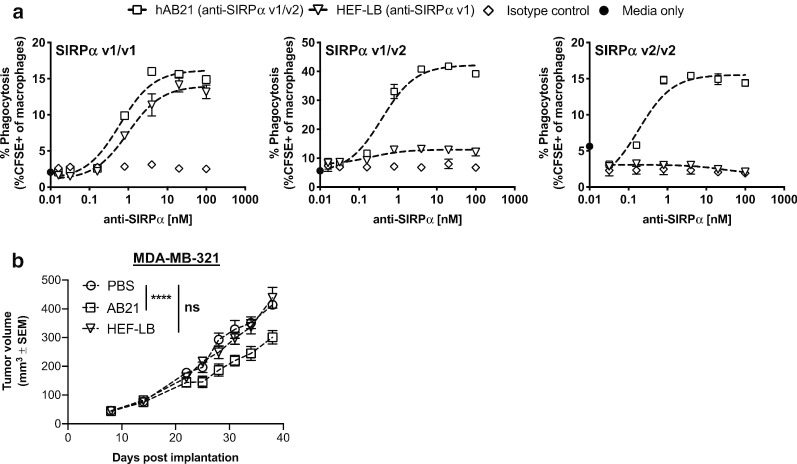
hAB21 potentiates macrophage-mediated ADCP and delays tumor growth in SIRPα v1/v2 humanized mouse model. **a** In vitro phagocytosis experiment with human M-CSF derived MDMs and DLD-1 cells in the presence of titrated hAB21, HEF-LB or isotype control (Fc inactive) antibodies. Percent of macrophages that engulfed CFSE-labelled tumor cells is indicated on the y-axis. On the y-axis, closed circle indicates background phagocytosis observed with media only. **b** MDA-MB-321 TNBC cells were implanted subcutaneously on the right flanks of humanized mice engrafted with human CD34+ cells from *SIRPA* v1/v2 donor. On day eight, mice with established tumors were randomized, *n* = 5 mice/group, and treated intratumorally with PBS, clone HEF-LB, and AB21 mIgG1_N297A. Mice were treated four times every 3 days. ****p < 0.0001, statistics were performed using Two-Way ANOVA, Tukey’s multiple comparisons test

Two allelic variants of SIRPα, v1 and v2, are present in the human population at various frequencies and zygosities dependent on ethnicity [22]. Antagonism of both SIRPα alleles is essential to ensure all population can be targeted to maximize the phagocytosis promoting activities of anti-SIRPα blocking antibodies [22, 32]. To investigate the influence of macrophage SIRPα genotype on the activity of hAB21, we derived human macrophages from primary human monocytes cultured in M-CSF (MDM), determined SIRPα v1 and v2 allelic status, and performed in vitro phagocytosis assays using v1/v1 homozygous, v1/v2 heterozygous, or v2/v2 homozygous MDMs as effectors. HAB21 potently enhanced macrophage phagocytosis of DLD-1 tumor cells in a dose-dependent manner irrespective of SIRPα genotype with an EC_50_ of ~ 0.2 nM, consistent with the high affinity binding of hAB21 to both SIRPα v1 and v2 variants (Fig. 3a). In contrast, the anti-SIRPα v1 specific antibody clone HEF-LB only promoted macrophage phagocytosis of DLD-1 cells by v1/v1 homozygous macrophages, with limited or no activity with v1/v2 heterozygous or v2/v2 homozygous macrophages, respectively (Fig. 3a). Similarly, in humanized mice engrafted with human CD34^+^ cells from donors with v1/v2 *SIRPA* genotype bearing MDA-MB-231 triple negative breast cancer (TNBC) tumors, AB21 significantly decreased antitumor activity while clone HEF-LB had no impact on tumor growth (Fig. 3b).

### HAB21 monotherapy modestly delays tumor growth and is dependent on DC-mediated anti-tumor CD8+ T cell response

**Fig. 4:**
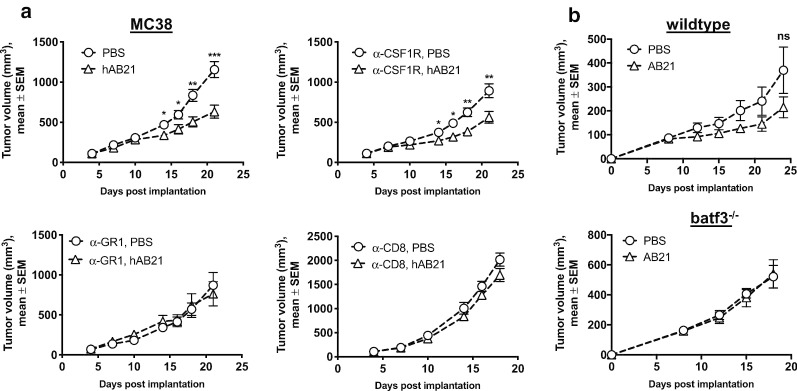
CD8^+^ T-cells, CD8^+^ DCs and granulocytes are necessary for in vivo efficacy of hAB21 monotherapy. **a** MC38 colon carcinoma cells were implanted subcutaneously in C57BL/6 mice. Two days post-implant, mice were treated with anti-CSF1R, anti-GR1 or anti-CD8 depleting antibodies. Mice with established tumors were randomized, *n* = 5 mice/group, and treated intraperitoneally with PBS or hAB21. Mice were treated three times every 3 days. Depleting antibodies were dosed on days 2, 5, 10 and 15 post-implantation. Unpaired two-tailed *t*-test between PBS and hAB21 were determined for days indicated. *p < 0.05, **p < 0.01, ***p < 0.001. **b** MC38 cells were implanted subcutaneously in wild type and Baft3-deficient C57BL/6 mice. Eight days post mice were randomized, *n* = 5 mice/group, and dosed intratumorally with vehicle or AB21. Mice were treated four times, every 3 days

**Fig. 5:**
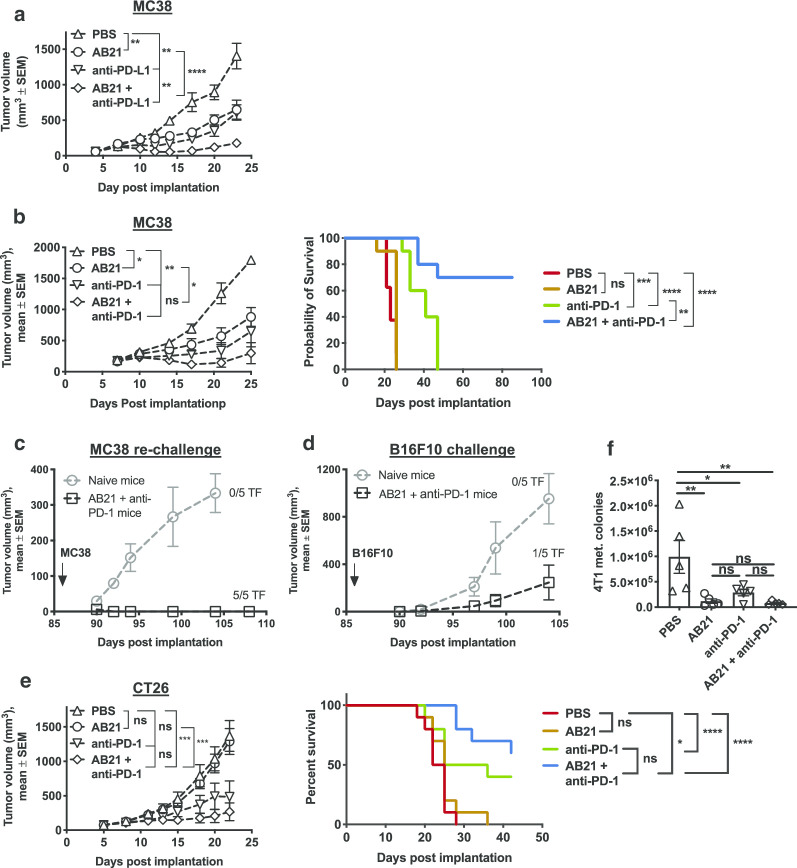
AB21 potentiates the antitumor activity of anti-PD-L1 and anti-PD1. MC38 colon carcinoma cells were implanted subcutaneously in C57BL/6 mice. Mice with established tumors were randomized and treated intraperitoneally. **a** Mice were treated with PBS, AB21, anti-PD-L1 or AB21 and anti-PD-L1, two times every week for 3 weeks. Graph shows tumor curve with *n* = 10 mice/group. Unpaired two-tail student’s *t*-test on day 17. **b** Mice were treated with PBS, AB21, anti-PD-1 or AB21 and anti-PD-1. Graphs show tumor growth of mice treated two times, every 3 days (left, unpaired two-tail *t*-test on day 25) and survival curves of mice treated three times every 3 days (Log-rank, Mantel–Cox test) of *n* = 10 mice. **c**, **d** MC38 tumor-bearing mice treated with AB21 + anti-PD-1 and achieved complete eradication were rechallenged with MC38 (**c**) and B16F10 (**d**) 60 days post-eradication. Concurrently, MC38 and B16F10 cells were implanted subcutaneously into age-matched naïve C57BL/6 mice. Graphs show tumor growth of MC38 and B16F10 tumors. **e** Mice bearing CT26 tumors were treated with PBS, AB21, anti-PD-1 or AB21 + ant-PD-1, four times, every 3 days. Graphs show tumor growth of *n* = 10 mice. Unpaired two-tail *t*-test on day 21 for growth curve and log-rank (Mantel–Cox) test for survival curves. **f** Total numbers of lung metastatic colonies in 4T1 tumor-bearing mice treated with PBS, AB21, anti-PD-1 or AB21 + anti-PD-1. The results are expressed as mean ± SD (*n* = 5). *p < 0.05, **p < 0.01, ***p < 0.001, ****p < 0.0001, ns indicates not significant

We determined the anti-tumor activity of hAB21 monotherapy in C57BL/6 mice bearing syngeneic MC38 tumors. Treatment with hAB21 alone produced a modest but statistically significant delay in tumor growth compared to the PBS control group (Fig. 4). Depletion of macrophages and myeloid cells (anti-CSF1R) had little to no impact on the anti-tumor activity of hAB21, whereas depletion of neutrophils (anti-GR1) and CD8^+^ (anti-CD8) T cells abrogated anti-tumor activity (Fig. 4) indicating a requirement for both innate and adaptive immunity. In batf3^−/−^ mice that lack CD8α^+^ DCs [3] treatment with hAB21 had no effect on MC38 tumor growth, indicating a requirement for CD8α^+^ DCs. Collectively, these results indicate that both innate and adaptive immune responses are crucial to the antitumor activity of hAB21 monotherapy, consistent with previous studies identifying both neutrophils and DCs as important immune effector cells responsible for the efficacy of anti-CD47 therapy in syngeneic tumor models [9, 13, 33]. Single agent activity of hAB21 in syngeneic models was restricted to MC38 tumors as we did not observe hAB21 activity in the more aggressive CT26, 4T1, or B16F10 tumors (Figs. 4, 5e, Additional file 1: Fig. S3).

### SIRPα antagonism improves the anti-tumor activity of T-cell checkpoint blockade in vivo

Since hAB21 monotherapy produced a modest antitumor response requiring both innate and adaptive immunity, we determined if hAB21 activity could be further enhanced by combination with T cell checkpoint antagonists targeting the PD-1/PD-L1 interaction. Indeed, combination therapy with AB21 and anti-PD-L1 significantly delayed MC38 tumor growth compared to AB21 or anti-PD-L1 alone (Fig. 5a). Combination of AB21 with anti-PD-1 also resulted in a significant delay in tumor growth, which translated to a significant extension of survival and complete regression of MC38 tumors in 60% (6/10) of mice, whereas there were no complete cures in single agent AB21 or anti-PD-1 treatment groups (Fig. 5b). All surviving AB21 and anti-PD-1 combination therapy treated mice were protected against subsequent re-challenge with MC38 tumor cells (Fig. 5c), whereas the same surviving mice challenged contralaterally with the unrelated B16F10 tumor were not protected (Fig. 5d). These results demonstrate the development of long-term, tumor-specific immune memory as a result of combination therapy. The growth of contralateral B16F10 tumors in mice surviving AB21 and anti-PD-1 combination therapy was delayed compared to age-matched naïve mice challenged with B16F10 (Fig. 5d), possibly due to the induction of a memory immune response against the MC38 tumor that may also respond to B16F10 tumor.

We further evaluated the efficacy of AB21 in combination with anti-PD-1 in BALB/c mice bearing subcutaneous CT26 tumors, a modestly immunogenic mouse colon carcinoma with variable responses to anti-PD-1 treatment [34]. Treatment with AB21 alone had no impact on tumor growth while treatment with anti-PD-1 alone trended toward a delayed tumor growth compared to the PBS control but did not reach statistical significance (Fig. 5e). Combination of anti-PD-1 with AB21 significantly delayed CT26 tumor growth compared to the PBS control and trended towards improved tumor control compared to the anti-PD-1 group (Fig. 5e).

The mouse 4T1 mammary tumor is poorly immunogenic, highly metastatic, and resistant to T cell checkpoint blockade [35]. BALB/c mice bearing subcutaneous 4T1 tumors were treated with AB21 and anti-PD-1 alone or in combination and primary tumor growth and metastatic burden in the lungs were quantified. Neither AB21 or anti-PD-1 given as a single agent or in combination had an effect on primary tumor growth (Additional file 1: Fig. S3A); however, AB21 monotherapy significantly reduced metastatic burden in the lung and a further reduction in metastatic burden in the AB21 and anti-PD-1 combination group, but did not reach statistical significance (Fig. 5f).

Finally, we evaluated the efficacy of AB21 in combination with an agonist antibody against the immune stimulatory molecule 4-1BB in C57BL/6 mice bearing subcutaneous B16F10 tumors, a poorly immunogenic, highly aggressive mouse melanoma that is resistant to anti-PD-1 therapy but partially responsive to anti-4-1BB [36]. Treatment with AB21 alone had no effect on B16F10 tumor growth, whereas anti-4-1BB significant delayed tumor growth compared to the PBS control (Additional file 1: Fig. S3B). Combination of anti-4-1BB with AB21 did not improve antitumor responses against B16F10 (Additional file 1: Fig. S3B). Collectively, the in vivo efficacy studies indicate that AB21 combines with ICIs targeting PD-1 or PD-L1 to improve tumor control that leads to durable antitumor immunity against MC-38 and CT26 tumors. In more aggressive and less immunogenic tumor types such as B16F10 and 4T1, responses to the combination of AB21 with anti-PD-1 or anti-4-1BB are less pronounced compared to responses in MC38 and CT26 tumor models.

### AB21 induces DC activation and promotes T cell effector functions in combination with anti-PD-1

**Fig. 6:**
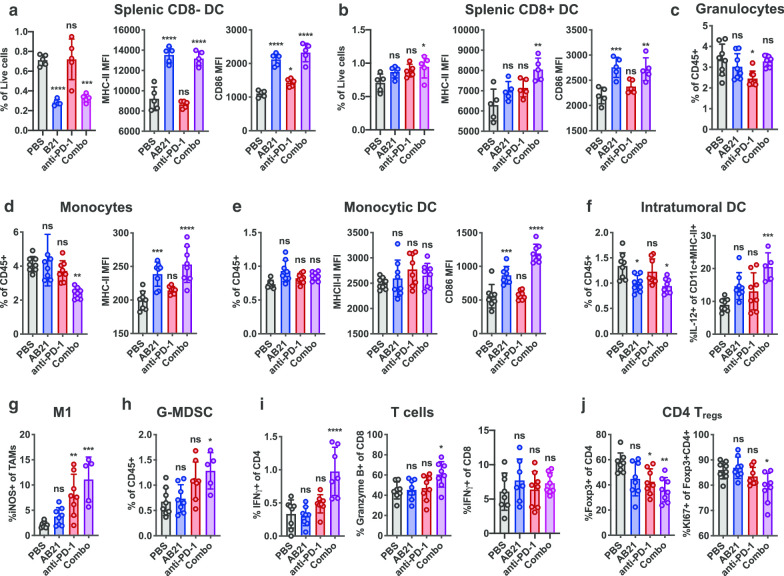
Characterization of the lymphoid and myeloid compartments of tumors and spleen. MC38 colon carcinoma cells were implanted subcutaneously in C57BL/6 mice. Mice with established tumors were randomized and treated intraperitoneally with PBS, AB21, anti-PD-1 or combo (AB21 + anti-PD-1). **a**, **b** Quantifications of splenic CD8^−^ and CD8^+^ CD11c^hi^MHCII^+^ DCs as a proportion of live cells and expression of costimulatory molecules in the spleen. **c**–**e** Quantification of granuloctyes (Gr1^mid^CD11b^+^), monocytes (Gr1^hi^CD11b^+^) and monocytic DCs (GR1^hi^CD11c^+^CD11b^+^MHC-II^+^) as a proportion of live CD45+ cells and expression of costimulatory molecules in the spleen. **f** Quantification of DCs (CD11c^+^MHC-II^+^) as a proportion of live CD45^+^ cells and IL-12^+^ DCs in the tumor. **g** Quantification of iNOS+ expressing cells as a proportion of total TAMs (CD11b^+^Gr1^−^MHC-II^+^) in the tumor. **h** Quantification of granulocytic monocyte derived suppressor cells, G-MDSCs, (CD11b^+^Gr1^mid^) as a proportion of live CD45^+^ cells in the tumor. **i** Quantification of IFNg^+^ cells as a proportion of CD4 and CD8 and Granzyme B as a proportion of CD8 in the tumor. **j** Quantification of Foxp3^+^ cells as a proportion of CD4 and Ki67^+^ cells as of Foxp3^+^CD4^+^ T_regs_ in the tumor. Plotted as mean ± SD and analyzed by Ordinary one-way ANOVA, Tukey’s multiple comparisons test. *p < 0.05, **p < 0.01, ***p < 0.001, ****p < 0.0001, ns is not significant

We conducted immunophenotyping using flow cytometry to directly access the cellular immune responses in MC38 tumor bearing mice treated with AB21 alone or in combination with anti-PD-1. MC38 tumor bearing mice were treated on days seven and ten with AB21 and one dose of anti-PD1 on day seven. Analysis of cellular responses in the spleen and tumor was performed 4 days post the final dose. Treatment with AB21 alone had little to no impact on the frequency of the various T cell subsets, NK cells, monocytes, CD8^+^DCs, or monocytic DCs (mDCs) in the spleen (Fig. 6, Additional file 1: Fig. S4A), but resulted in a significant reduction of CD8^−^ DCs concomitant with an upregulation of the activation markers MHC class II and CD86 (Fig. 6a). The combination of AB21 and anti-PD-1 also activated and reduced the frequency of CD8^−^ DCs in the spleen, whereas treatment with anti-PD-1 alone had no effect (Fig. 6a) indicating that DC activation is a result of SIRPα blockade with AB21. CD8^+^ DCs in the spleens of mice treated with AB21 alone or in combination with anti-PD-1 also displayed an activated phenotype and combination therapy resulted in a small but significant increase in the frequency of CD8^+^ DCs in the spleen (Fig. 6b). Furthermore, monocytes and mDCs were activated upon treatment with AB21 either alone or in combination with anti-PD-1 (Fig. 6d, e). Activation by AB21 was associated with a decrease in monocyte frequency and no change in mDC frequency in the spleen (Fig. 6d, e).

Within the tumor microenvironment, AB21 monotherapy and combination therapy with anti-PD-1 caused a slight reduction in CD11c^+^MHC-II^+^ DCs; however, DC production of IL-12, a marker of activated DCs and a potent proinflammatory cytokine necessary for tumor control in response to ICIs [37], was significantly increased in response to combination therapy but not AB21 or anti-PD-1 monotherapy (Fig. 6f). Tumor-associated macrophages (TAMs) account for the majority of leukocytes within MC38 tumors and treatment with AB21, anti-PD-1, or the combination had no effect on TAM frequency (Additional file 1: Fig. S4B); however, iNOS production, a marker of M1, was significantly elevated in both the anti-PD-1 monotherapy and combination therapy groups (Fig. 6g). G-MDSC infiltration increased upon treatment with the combination of AB21 and anti-PD-1 (Fig. 6h). Additionally, surface expression of SIRPα on splenic and tumor myeloid cells was downregulated in response to AB21 treatment (both alone and in combination with anti-PD-1), but not in the anti-PD1 treatment group (Additional file 1: Fig. S4C). Downregulation of SIRPα by AB21 provides an additional mechanism of disrupting CD47–SIRPα signaling which likely contributes to the antitumor activity of AB21.

Potentiation of innate immune cell functions by AB21 facilitated further improvements in T effector cell responses to anti-PD-1 therapy with no changes to cell frequency (Additional file 1: Fig. S4D). Combination therapy promoted IFNγ production by CD4^+^ T cells in the tumor and CD8^+^ T cells in the spleen and granzyme B production by CD8^+^ T cells in the tumor (Fig. 6i, Additional file 1: S4A). In contrast monotherapy had limited impact on T cell effector functions. Furthermore, combination therapy significantly reduced the number of CD4^+^ regulatory T cells (T_regs_) in the tumor and decreased their proliferative capacity (Fig. 6j). The cellular responses to AB21 treatment are broadly in agreement with the cell depletion studies indicating both DCs and CD8^+^ T cells are indispensable for tumor control (Fig. 5). Collectively, these data indicate that SIRPα blockade with AB21 results in the repolarization of the myeloid-compartment, as evident by the increased activation phenotype and IL-12 production of the DCs as well as increased iNOS producing M1-like TAMs, resulting in an anti-tumorigenic phenotype.

### HAB21 demonstrates favorable PK, PD and tolerability profiles in monkeys

**Fig. 7:**
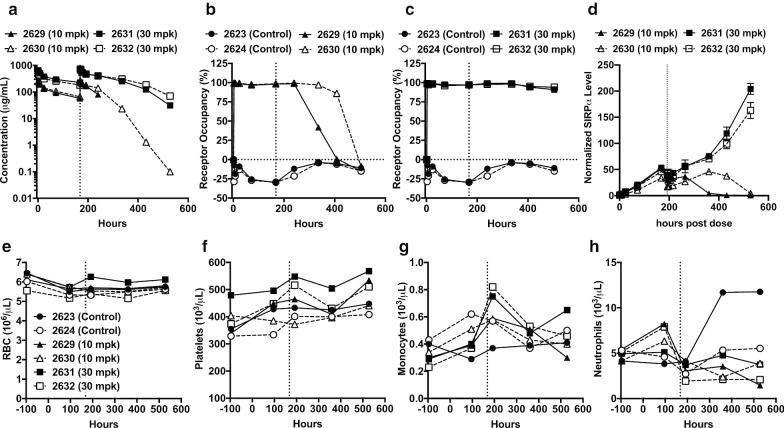
Pharmacokinetic and safety evaluation of hAB21 in cynomolgus monkey. Female cynomolgus monkeys were dosed twice, 1 week apart, with PBS (control), 10 or 30 mpk hAB21-Fc^dead^. Serum concentration of hAB21 (**a**) and occupancy of SIRPα on monocytes at the indicated time points in monkeys administered 10 mpk (**b**) and 30 mpk (**c**). Increase in soluble SIRPα over time in monkeys dosed with 10 or 30 mpk (**d**). Vertical dashed lines indicated infusion of monkey on day 8. **e**–**h** Hematology parameters of RBC (**e**), platelets (**f**), monocytes (**g**) and neutrophils (**h**)

HAB21 binds to both cynomolgus monkey SIRPα and human SIRPα with similar affinity (Fig. 1). Therefore, we conducted an exploratory study in cynomolgus monkey to determine the PK, PD and preclinical safety of hAB21. Monkeys received two weekly injections of vehicle control or hAB21-IgG1^FcDead^ on days zero and seven at doses of either 10 mg/kg or 30 mg/kg and total serum antibody concentration, receptor occupancy, hematologic parameters and serum chemistry were determined. Systemic exposure to hAB21 increased linearly from the 10 to 30 mg/kg dose level with an AUC_0168_ of 17,783 ng h/mL and 48,635 ng h/mL, a clearance of 0.35 mL/hr/kg and 0.29 mL/h/kg, and a half-life of 5.3 days and 8.1 days, respectively (Fig. 7a). Complete target occupancy was observed in circulating monocytes within 3 h of dosing at both dose levels that persisted for 7 days post the first dose (Fig. 7b, c) and 14 days post the second dose at the 30 mg/kg dose (Fig. 7c). Accelerated clearance and the concomitant loss of target occupancy were observed in monkeys that received the 10 mg/kg dose (Fig. 7b) at time points on Day 14 and beyond, suggestive of an anti-drug antibody (ADA) response to human proteins.

Proteolysis and release of soluble SIRPα from cell-surface expression has been demonstrated in cultured cells [38]. To investigate the presence of soluble SIRPα and potential impact of soluble SIRPα on PK of anti-SIRPα antibodies in the monkey studies, we used non-cross blocking antibodies to capture and measure free and antibody-bound soluble SIRPα in the serum. In all dosed monkeys, soluble SIRPα was detected to be present and accumulates during the course of the study as compared to predose levels (Fig. 7d). There is a 50-fold increase in soluble SIRPα as compared to predose after the first dose at both 10 and 30 mg/kg. Post the second dose, the fold increase in soluble SIRPα reaches 200-fold at the 30 mg/kg dose and back to low levels by 14 and 21 days at the 10 mg/kg dose (Fig. 7d). The concentration of soluble SIRPα correlates with the PK of hAB21 (Fig. 7a), indicating accumulation of circulating soluble SIRPα due to decrease clearance while bound to hAB21.

No adverse findings for clinical signs, hematology and serum chemistry related to SIRPα blockade by hAB21 were observed at either dose level (Fig. 7e–h). No significant changes in red blood cells (Fig. 7e) and platelets (Fig. 7f) were observed. In addition, monocytes and neutrophils, which express high levels of SIRPα, remained unaffected by hAB21 treatment (Fig. 7g, h). Collectively the data indicate that hAB21 is safe and well-tolerated in cynomolgus monkeys at repeated doses up to 30 mg/kg with linear PK and full target occupancy in blood.

## Discussion

Blockade of the CD47–SIRPα interaction represents a promising approach to boost the antitumor activity of cancer immunotherapies when used as an adjuvant in antitumor antibody or ICI therapy. A variety of CD47 blocking antibodies or Fc-fusion proteins have shown objective responses in patients with advanced hematologic or solid tumor malignancies [14–17, 39, 40]. While a multitude of agents that target CD47 have been investigated in pre-clinical models and clinical trials, only a limited number of anti-SIRPα antibodies have been reported [32, 41]. However, due to the lack of species cross-reactivity, no anti-SIRPα antibody has, to our knowledge, been investigated in both mouse and monkey to evaluate safety and efficacy. The development of antibody-based SIRPα antagonists suitable for clinical translation has been hindered in part by polymorphisms within the CD47-binding domain of SIRPα, which necessitates pan-allele reactive anti-SIRPα antibodies for therapeutic intervention in diverse patient populations. Therefore, the impact of targeting SIRPα compared to CD47 was investigated as differences in target expression profile and function may result in divergent activities of anti-CD47 versus anti-SIRPα targeted therapies that could yield potentially distinct pharmacokinetic and safety profiles.

We previously identified anti-SIRPα antibodies from immunized chickens, which are phylogenetically distant from human, monkey, and mouse, which enabled the discovery of a diverse array of pan-allelic and species cross-reactive anti-SIRPα antibodies [22]. Herein, we selected the anti-SIRPα antibody clone AB21 for humanization and further functional characterization due to its high affinity binding to both human SIRPα v1 and v2 alleles, comparable binding to cynomolgus monkey SIRPα, cross-reactivity with various mouse SIRPα alleles, and ability to potently block the interaction between CD47 and SIRPα. Allele cross-reactivity is an important design criterion for anti-SIRPα antibodies intended for clinical use. Several groups have reported the presence of multiple SIRPα alleles in the human population [32, 42]. However, in our analyses of 2535 individuals from 1000 Genome Project, we determined that only two dominant SIRPα alleles, v1 and v2, are present at various frequencies dependent on ethnicity [22]. Blockade of both SIRPα alleles is necessary to maximize efficacy; a single functional SIRPα variant can compensate for the antagonized variant. As demonstrated in Fig. 3, the v1 allele-specific anti-SIRPα antibody HEF-LB enhanced macrophage-mediated ADCP only when v1/v1 homozygous macrophages were used as effectors. HEF-LB has little to no impact on ADCP mediated by v1/v2 heterozygous or v2/v2 homozygous macrophages and on antitumor immunity by v1/v2 heterozygous donor (Fig. 3). In contrast, hAB21 is efficacious independent of SIRPα genotype (Fig. 3). SIRPα v1 specific antibodies, such as HEF-LB, would be limited to use in v1/v1 homozygote patient populations, which ranges from only 13.3–49.1% frequency among the global sub-populations [22]. Pan-allelic antibodies such as hAB21 can potentially impact the entire population, negating the need to stratify patients prior to therapy, a desirable property for clinical translation.

In addition to species and allele cross-reactivity, the choice of IgG subclass is an important consideration when designing therapeutic antibodies. The efficacy of both antitumor and immunomodulatory antibodies is highly dependent on Fc-domain engagement of FcγRs [43–45]. FcγR interactions with tumor antigen specific antibodies are crucial for the induction of antitumor immunity [45] but can be detrimental to the activity of immunomodulatory antibodies due to unwanted depletion of antigen-positive effector cells [43]. Toxicity may also result from FcγR interactions that promote antibody-mediated destruction of undesirable cell types and tissues, as is the case for CD47-targeted therapies capable of engaging activating FcγRs [20]. CD47 is expressed broadly, including on RBCs and platelets. Hematological toxicities including thrombocytopenia and anemia are observed in patients treated with anti-CD47 antibodies capable of triggering FcγR-dependent effector functions [19, 20, 39]. We have previously demonstrated that safety liabilities associated with targeting CD47 can be mitigated by eliminating binding to FcγRs, leading to the clinical development of ALX148 [13], a fusion protein consisting of a high-affinity SIRPα variant fused to an inactive Fc-domain [46].

To investigate the role of FcγR interactions on anti-SIRPα antibody activity, we substituted the Fc-domain of hAB21 with various natural or engineered IgG Fc-domains that have differential binding to FcγRs. Inhibiting the CD47–SIRPα interaction generally enhances macrophage-mediated ADCP of tumor cells. However, we found that potentiation of ADCP was highly dependent on the hAB21 Fc-domain; hAB21 with active Fc-domains (IgG1 and IgG4) failed to enhance ADCP, whereas hAB21 with inactive Fc-domains potentiated ADCP (Fig. 2a). This differential activity was demonstrated to be due to competition between the antitumor antibody cetuximab and hAB21 for binding to effector cell FcγR. Our data corroborate Voets et al. [32] showing abrogation of rituximab induced phagocytosis when anti-SIRPα is expressed with an active Fc. Interestingly, DCs numbers were reduced significantly in PBMCs cultured in vitro with hAB21 containing active Fc-domains, whereas hAB21 with an inactive Fc-domain had no effect on DC numbers in PBMC cultures (Fig. 2b). Monocytes, despite expressing high levels of SIRPα, were unaffected by hAB21 irrespective of IgG subclass (Additional file 1: Fig. S1). SIRPα expression on macrophages and DCs is downregulated by hAB21 (Additional file 1: Fig. S4C) and SIRPα downregulation can reduce SIRPα tonic signaling resulting in DC activation, maturation, and turnover, a mechanism previously reported in mice treated with an anti-SIRPα antibody [47, 48] and similar to what we observed in mice treated with hAB21 (Fig. 6). SIRPα downregulation by hAB21 may contribute to the DC depleting effect of hAB21; however, all anti-SIRPα antibodies regardless of subclass downregulate SIRPα but only hAB21 with active Fc-domains deplete DCs. We speculate that the DC depleting effect of hAB21 with an active Fc-domain is due to monocyte- and/or NK cell-mediated killing of DCs opsonized with hAB21; however, we cannot rule out that the combination of SIRPα downregulation and FcγR signaling induced by hAB21 with an active Fc-domain contributes to DC maturation and subsequent apoptosis.

A similar relationship between FcγR-binding and hAB21 activity was observed in vivo. hAB21 with a mouse IgG2a Fc-domain, which preferentially binds activating FcγRs, reduced the antitumor activity of rituximab whereas hAB21 with a mouse IgG1-N297A Fc-domain (with limited FcγR binding) enhanced rituximab antitumor activity. Although we did not investigate the in vivo mechanism responsible for these divergent activities, based on our in vitro results it is likely due to “scorpion effect”, competition between rituximab and AB21 for binding to effector cell FcγR, unwanted depletion of DCs, or all. Thus, similar to CD47-targeted therapies, ablation of FcγR-dependent effector function is desirable when targeting SIRPα, albeit for distinct reasons. Eliminating Fc-effector function from CD47 targeted therapies mitigates cytopenia [13], while eliminating effector function from anti-SIRPα antibodies is necessary to enhance macrophage-mediated ADCP and to prevent DC depletion in vitro.

We and others have demonstrated that blocking the CD47–SIRPα interaction has a profound impact on both antitumor antibody therapy and T cell checkpoint blockade, resulting in a coordinated innate and adaptive antitumor immune response mediated by multiple cell types including macrophages, dendritic cells, neutrophils, and T cells [9, 13, 41, 49, 50]. In most reported studies the target is CD47, and little is known about the in vivo pharmacology of targeting SIRPα. Therefore, we investigated the impact of SIRPα antagonism on antitumor immunity in mouse tumor models using hAB21 as a monotherapy or in combination with an antitumor antibody or T cell checkpoint inhibitors. We selected hAB21 containing an inactive Fc-domain for all in vivo studies due to the impact of Fc-effector function on hAB21 activity in vitro. Similar to targeting CD47 [13, 31], SIRPα blockade with hAB21 improved responses to rituximab (anti-CD20) in the Raji human tumor xenograft model, indicating that a major mechanism of action of both anti-CD47 and anti-SIRPα therapies in xenograft models, which lack NK, B and T cells, is to enhance the antitumor activity of myeloid cells. Although we did not investigate the effector cells responsible for hAB21-mediated tumor eradication in the Raji xenograft model, Ring et al. demonstrated that neutrophils and macrophages are the main effector cells responsible for the anti-tumor activity of a SIRPα blocking antibody in human tumor xenograft models [41]. These data are consistent with a large body of evidence implicating phagocytes as the dominant effector cell responsible for the efficacy of agents that block the CD47–SIRPα interaction in xenograft models [31, 46, 51].

Disrupting the CD47–SIRPα interaction bridges innate and adaptive antitumor immune mechanisms [9, 13]; therefore, we determined the activity of hAB21 in multiple syngeneic murine tumor models as a monotherapy or in combination with immune checkpoint inhibitors. These models encompass various levels of immunogenicity and sensitivity towards T cell checkpoint inhibition. As monotherapy, interfering with the CD47–SIRPα interaction generally has limited efficacy but is a powerful adjuvant to a variety of cancer immunotherapies [31, 46]. Treatment with hAB21 alone had no effect on tumor growth in syngeneic 4T1, CT26, or B16F10 tumors (Fig. 5e and Additional file 1: Fig. S3). Modest hAB21 single agent activity was observed against MC38 tumors (Figs. 4a, 5a, b), which was dependent on the presence of CD8α^+^ DCs, CD8^+^ T cells and neutrophils (Fig. 4). Depletion of neutrophils by anti-GR1 but not macrophages by anti-CSF1R prior to hAB21 treatment in immunocompetent mice showed no antitumor activity, suggesting cell depletion by anti-GR1 impacted hAB21-induced antitumor activity. Although, anti-GR1 has been used extensively to deplete neutrophils in C57BL/6 mice, it does also bind GR1+^+^ non-neutrophils, specifically CD8^+^GR1^+^ T cells [52, 53]. The lack of antitumor activity with anti-GR1 depletion may be due to the elimination of CD8^+^GR1^+^ T cells, known to secrete IFNγ, neutrophils or both cell types. CD8α^+^ DCs are indispensable for the production of a CD8^+^ antitumor T cell response [3] and DCs play a critical role in the responses to anti-CD47 targeted therapies [9, 13, 33]. Administration of hAB21 to MC38 tumor bearing mice resulted in DC activation in the spleen and tumor that when further combined with anti-PD-1/PD-L1 therapy promoted both CD4+^+^ and CD8^+^ T cell effector functions and reduced immunosuppressive CD4^+^ T_regs_ and TAMs leading to eradication of MC38 tumors and long-term, durable immunity. Similar mechanisms of MC38 tumor control have been reported for anti-CD47 antibodies [33, 54], and for ALX148, a CD47 blocking Fc-fusion protein [13], suggesting that the antitumor effects of targeting SIRPα broadly recapitulate that of CD47-targeted therapies despite differences in the ligand expression patterns and molecular and cellular functions of SIRPα and CD47.

Responses to the combination of hAB21 with anti-PD-1 or anti-4-1BB were less pronounced in more aggressive and less immunogenic tumors including CT26, 4T1, and B16F10 (Additional file 1: Fig S3). These results are consistent with additional studies that have investigated the combination of CD47 antagonists with checkpoint regulators in syngeneic mouse tumors [49]. In more aggressive and poorly immunogenic tumors, additional treatment modalities may be necessary to improve tumor control and eradication, such as triple combinations with tumor antigen specific antibodies, CD47/SIRPα antagonists, ICIs, vaccines, or chemotherapy [49, 55].

Due to the widespread expression of CD47, targeting SIRPα may overcome some obstacles associated with targeting CD47 [32, 56] such as the large antigen sink that promotes target mediated clearance of anti-CD47 therapies and off-tumor toxicities due to CD47 expression on RBCs and platelets by certain anti-CD47 therapeutics. We investigated safety and PK/PD of hAB21 in an exploratory toxicology study in cynomolgus monkey. No dose-dependent adverse clinical observations or changes in hematology and serum chemistry assessment were observed with hAB21 treatment (Fig. 7), whereas anti-CD47 therapeutics capable of activating FcγR-dependent effector functions causes dose-dependent cytopenia in NHPs and humans [19, 20]. Administration of hAB21 with an inactive Fc at both 10 mg/kg or 30 mg/kg resulted in complete target occupancy on circulating monocytes and linear PK with a half-life of 5.3 days and 8.1 days, respectively. This contrasts with anti-CD47 antibodies or anti-CD47 Fc-fusion proteins which display non-linear PK and accelerated clearance at similar dose levels due to the large CD47 antigen sink [20]. However, soluble SIRPα was detected in the monkeys and accumulation observed during the course of the study (Fig. 7d). This is indicative of serum stabilization of the naturally produced soluble SIRPα by binding to the anti-SIRPα antibody. Soluble SIRPα-bound antibody is not available for blocking cellular CD47–SIRPα interaction, potentially limiting the impact of the longer half-life of anti-SIRPα antibodies in comparison to anti-CD47 antibodies.

## Conclusion

In summary, blockade of SIRPα with hAB21 potently enhances macrophage phagocytosis of tumor cells and promotes activation of DCs leading to the induction of adaptive antitumor T cell responses in tumor bearing mice, substantially improving responses to both antitumor antibody and immune checkpoint therapy. The in vitro and in vivo antitumor activity of hAB21 broadly recapitulates that of CD47 targeted therapies despite differences in ligand expression, binding partners, and function, validating the CD47–SIRPα axis as fundamental myeloid checkpoint pathway and its blockade as promising therapeutic intervention for treatment of human malignancies.

## Supplementary information

**Additional file 1.** Supplementary figures and table.

## Data Availability

All relevant data are within the paper and its Supplementary files. Structure information is provided in Protein Data Bank (PDB).

## Abbreviations

ADA: Anti-drug antibody
ADCP: Antibody-dependent cellular phagocytosis
DC: Dendritic cell
FcγR: Fragment crystallizable gamma receptor
ICI: Immune checkpoint inhibitor
mDC: Monocytic dendritic cell
MDSC: Myeloid-derived suppressor cell
PBMC: Peripheral blood mononuclear cell
PK: Pharmacokinetic
SIRPα: Signal regulatory protein
TAM: Tumor-associated macrophage
TNBC: Triple negative breast cancer
Treg: Regulatory T cell
