# Supplementary material for: Targeting the myeloid checkpoint receptor SIRPα potentiates innate and adaptive immune responses to promote anti-tumor activity
Source: J Hematol Oncol. 2020 Nov 30;13:160. doi: 10.1186/s13045-020-00989-w (PMC7706287; doi:10.1186/s13045-020-00989-w)
Supplement: Supplementary file 1 — Additional file 1. Supplementary figures and table. [file 13045_2020_989_MOESM1_ESM.pdf]

Supplementary Figures

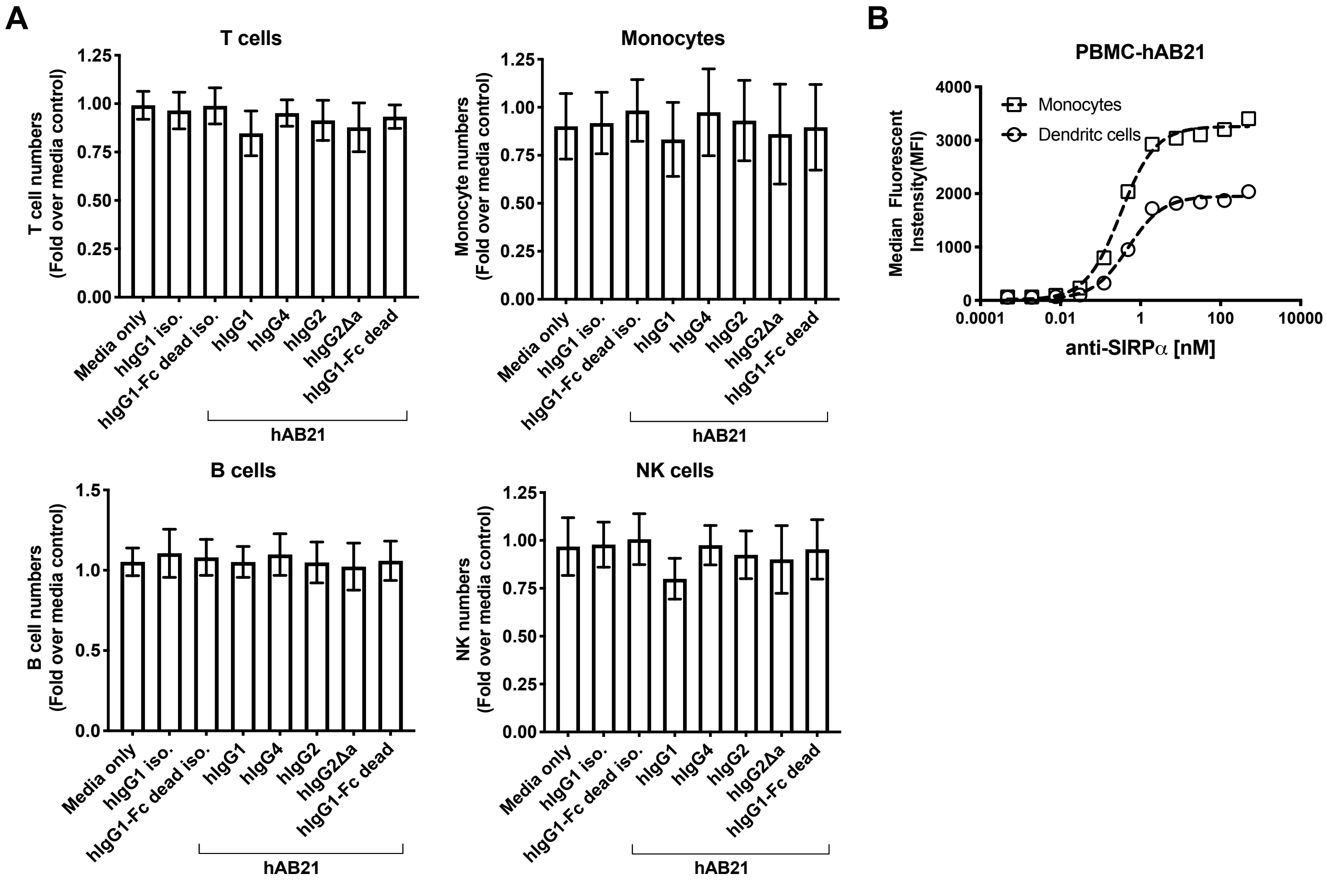

Figure S1. IgG subclasses show no effect on monocytes, B, T and NK cells. (A) Relative monocytes, B, T, and NK cell numbers in human PBMCs after 48 hour incubation with hAB21-IgG subclass variants. Each bar indicates relative cell subset number as compared to media only. IgG control is abbreviated as iso. (B) Flow cytometry analysis of hAB21 binding to human CD14$^+$ monocytes and CD11c$^+$MHCII+ DCs. Fluorescent-labeled hAB21 was incubated in increasing concentration with PBMCs at 4˚C for 60 minutes, washed and evaluated by flow cytometry. Mean fluorescent intensity is measured.

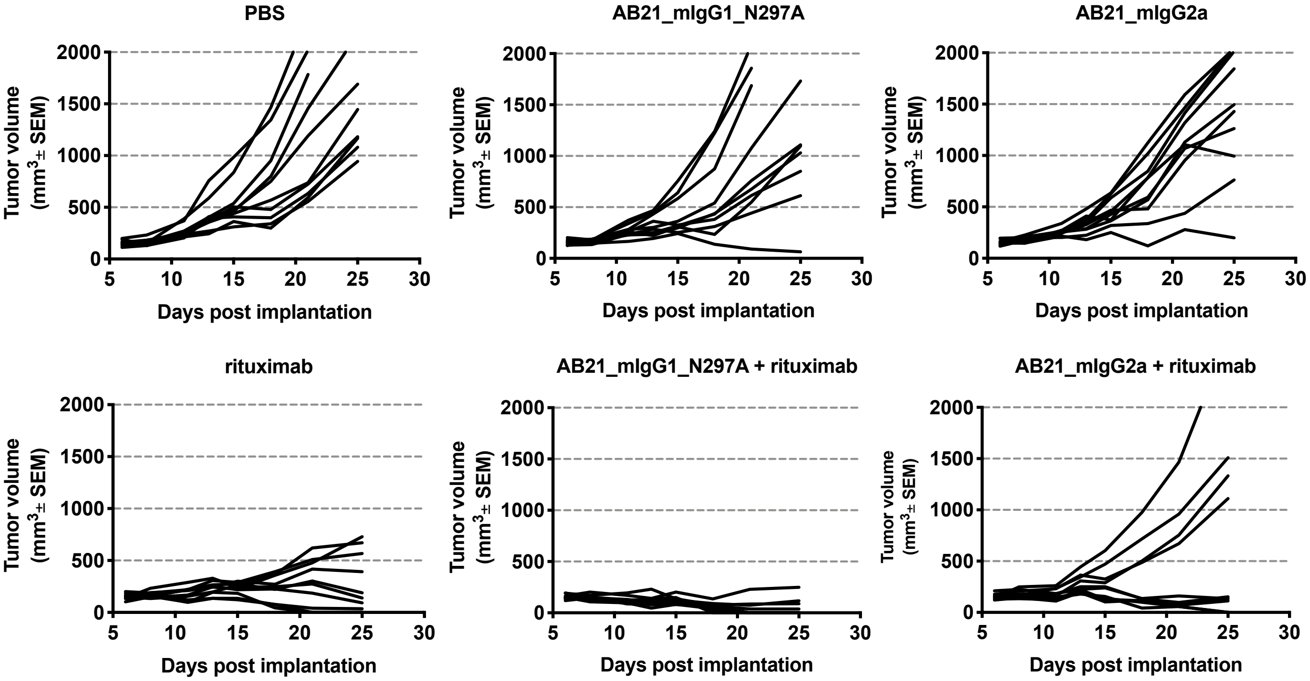

Figure S2. AB21-Fc-inactive is superior to AB21-Fc-active in combination with rituximab *in vivo.* Raji B-cell lymphoma cells were implanted subcutaneously on the right flanks of NOD-SCID mice. Mice with established tumors (average of 154 mm^3) were randomized, n=10/group, and treated intraperitoneally with vehicle, rituximab, AB21-Fc mIgG1_N297A (inactive), AB21-Fc h21_mIgG2a (active), or AB21-Fc inactive or –Fc active + rituximab. Mice were treated five doses every 3 days. Each line represents one mouse.

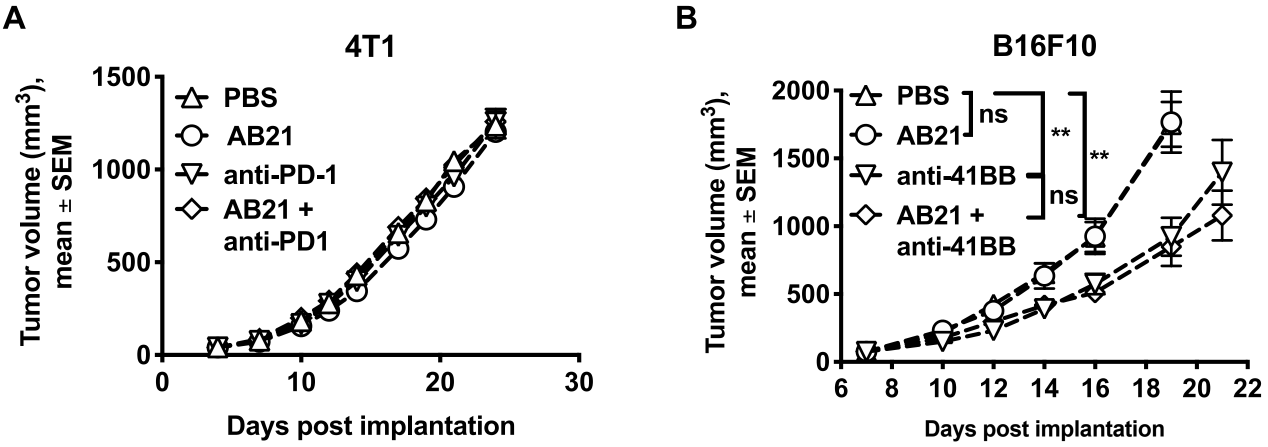

Figure S3. Single and combination efficacy in less immunogenic models, B16F10 and 4T1. (A) 4T1 cells were implanted subcutaneously in Bab/c mice Mice with established tumors were randomized, n=10 mice/group, and treated intraperitoneally with PBS, AB21, anti-PD-1, or AB21 + anti-PD-1. Mice were treated four times every three days for anti-PD-1 and five times every three days for AB21. **(B**) B16F10 melanoma cells were implanted subcutaneously in C57BL/6 mice. Mice with established tumors were randomized, n=10 mice/group, and treated intraperitoneally with PBS, AB21, anti-41BB, or AB21 + anti-41BB. Mice were treated three times every three days. One-Way ANOVA Tukey's multiple comparisons test on D19 was performed. **p<0.01 and ns is not significant

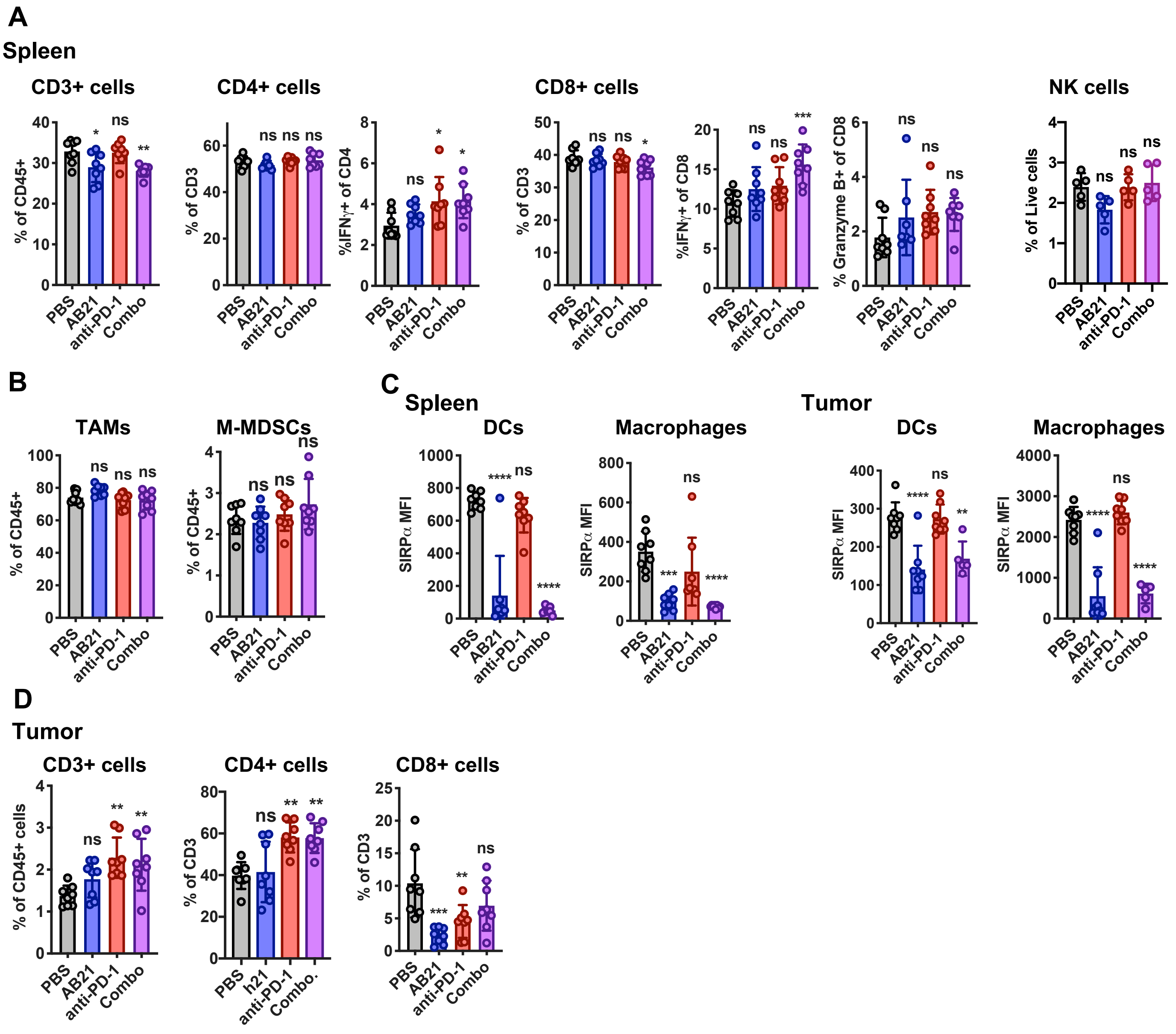

Figure S4. Characterization of the lymphoid and myeloid compartments of tumors and spleen. MC38 colon carcinoma cells were implanted subcutaneously in C57BL/6 mice. Mice with established tumors were randomized and treated intraperitoneally with PBS, AB21, anti-PD-1 or combo (AB21 + anti-PD-1). (A) Quantifications of splenic CD3, CD4, CD8 and NK cells as a proportion of CD45 or CD3 and IFNγ and granzyme B expressing T cells. (B) Quantifications of tumor associated macrophages (TAMs) and M-MDSCs in tumors. (C) Quantification of SIRPα expression on monocytes ($Gr1^{hi}CD11b^{+}$) and DCs ($CD11c^{+}MHC\text{-}II^{+}$) in spleen and tumor. (D) Quantification of CD3, CD4 and CD8 as a proportion of live $CD45^{+}$ or $CD3^{+}$ cells in the tumor. Plotted as mean +/- SD and analyzed by Ordinary one-way ANOVA, Tukey's multiple comparisons test. *p < 0.05, **p < 0.01, ****p < 0.0001, n.s. is not significant.

Table S1

**Data Collection**	
Space group	P6122
Cell dimensions	
a, b, c (Å)	189.1, 189.1, 78.4
α, β, γ (Å)	90.00, 90.00, 120.00
Resolution	61.92 – 2.27 (2.32 – 2.27)
Unique Reflections	38395 (2803)
I/σI	10.9 (1.6)
Completeness (%)	99.6 (99.9)
Redundancy	13.5 (13.8)
R_{sym}	17.6 (173.1)
**Refinement**	
Resolution (Å)	61.92 – 2.27 (2.32 – 2.27)
No. reflections	
Used for refinement	36422
Used for Rfree calculation	1972
R_{factor} (%)	23.6 (32.7)
R_{free} (%)	28.1 (36.7)
No. atoms	
Protein	4050
Water molecules	166
Sulfates	5
B-factors (Å2, average)	
Protein	40.51
Water molecules	36.28
r.m.s. deviations	
Bond lengths (Å)	0.0097
Bond angles (º)	1.4812
Ramachandran Favored (%)	95.67
Ramachandran Allowed (%)	5.16
Ramachandran Outliers (%)	0.94

*Values in parentheses are for the highest resolution shell.
